# Effect of Pregnancy on Ventricular and Aortic Dimensions in Repaired Tetralogy of Fallot

**DOI:** 10.1161/JAHA.116.005420

**Published:** 2017-07-23

**Authors:** Matthew Cauldwell, Michael A. Quail, Gillian S. Smith, Ee Ling Heng, Sarah Ghonim, Anselm Uebing, Lorna Swan, Wei Li, Roshni R. Patel, Dudley J. Pennell, Philip J. Steer, Mark R. Johnson, Michael A. Gatzoulis, Sonya V. Babu‐Narayan

**Affiliations:** ^1^ Academic Department of Obstetrics and Gynaecology Imperial College London United Kingdom; ^2^ Adult Congenital Heart Centre The National Heart and Lung Institute Imperial College London United Kingdom; ^3^ NIHR Cardiovascular Biomedical Research Unit Royal Brompton Hospital Imperial College London United Kingdom; ^4^ Chelsea and Westminster Hospital London United Kingdom

**Keywords:** cardiovascular magnetic resonance imaging, pregnancy, tetralogy of Fallot, Magnetic Resonance Imaging (MRI), Remodeling, Congenital Heart Disease

## Abstract

**Background:**

The aim was to assess whether cardiovascular adaptation to pregnancy in women with repaired tetralogy of Fallot (TOF) adversely affects hemodynamic stability, in particular with respect to right ventricular (RV) dilatation, pulmonary regurgitation, or aortic root dilatation.

**Methods and Results:**

This was a retrospective cohort study of women with repaired TOF with paired cardiovascular magnetic resonance scans before and after their first pregnancy (baseline RV end systolic volume index 49 mL/m^2^ and RV end diastolic volume index 118 mL/m^2^) matched with a comparison group of nulliparous women with TOF. Cases were matched for age at baseline cardiovascular magnetic resonance scan, time between follow‐up of cardiovascular magnetic resonance scans, QRS duration, RV ejection fraction, and indexed RV end systolic and diastolic volume at baseline. Effect of pregnancy and time on parameters was assessed using mixed‐effects modelling. Nineteen women with repaired TOF who had completed their first pregnancy were identified and matched with 38 nulliparous women. We observed no deleterious effects of pregnancy on RV volumes, aortic dimensions, or exercise data. There was an effect of pregnancy observed in both left ventricular end diastolic volume and left ventricular stroke volume, consistent with a sustained small increase in left ventricular stroke volume attributed to pregnancy (53–55 mL/m^2^).

**Conclusions:**

Women with repaired TOF and with mild‐to‐moderate RV dilatation considering pregnancy can be reassured that pregnancy is unlikely to cause deterioration in their cardiovascular status. We recommend that women are routinely assessed and followed up before and after pregnancy and that prepregnancy counseling is tailored to their individual clinical status.

**Clinical Perspective**

**What is New?**

- In women with mild to moderate right ventricular dilatation, pregnancy is not associated with significant adverse right ventricular remodeling or an increase in aortic dimensions.

**What are the Clinical Implications?**

- In preconception counseling for patients with characteristics similar to the study group, women can be advised that pregnancy is unlikely to accelerate progressive right ventricular disease.
- Given the low risks associated with pregnancy in women with mild to moderate right ventricular dilatation, pulmonary valve replacement on the basis of a future pregnancy alone is unlikely to be justified if right ventricular volumes are below current thresholds.

## Introduction

Long‐term survival following tetralogy of Fallot (TOF) repair is excellent, with survival at 30 years postsurgery approaching 90%.^1^ However, increased morbidity and mortality, commencing particularly from the third postoperative decade, coincides with a time when many women will consider becoming pregnant. Approximately 10% of women with repaired TOF experience short‐term cardiovascular complications during pregnancy, predominately supraventricular arrhythmias.^2^ However, the longer‐term effects of pregnancy have been less well studied.

Normal cardiovascular adaptation to pregnancy is characterized by marked increases in cardiac output and total circulating volume with corresponding decreases in systemic vascular resistance.^3^ There is concern that these hemodynamic changes may adversely affect cardiac structure and function in patients with repaired TOF. This view is supported by previous imaging studies indicating persistent and progressive right ventricular (RV) dilatation as a consequence of pregnancy.^4^, ^5^ There is also concern about pregnancy‐associated changes in the aortic root, but no data currently exist.^6^, ^7^

Contemporary studies are limited to small numbers of women with more‐severe baseline RV dilatation. Therefore, pregnancy counseling in this group may be negatively biased.

In this study, our aim was to determine whether pregnancy is associated with a significant cardiovascular deterioration in a contemporary cohort of women with mild‐to‐moderate RV dilatation. We assessed both RV and left ventricular (LV) volumes and aortic dimensions in a group of patients before and at least 6 months after pregnancy using cardiovascular magnetic resonance (CMR). We compared them with nonpregnant women with repaired TOF matched according to baseline characteristics and who were imaged over a similar time interval.

## Methods

**Figure 1:**
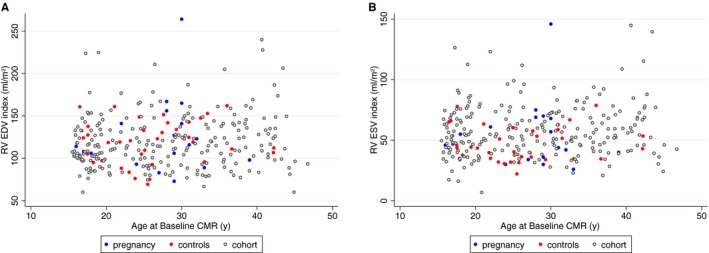
RV EDV index (A) and RV ESV index (B) in all women of childbearing age with repaired tetralogy of Fallot in our institution at age of first CMR. The figure identifies those women who became pregnant and are included in this study (blue) and matched controls (red) from the cohort (gray). CMR indicates cardiovascular magnetic resonance; EDV, end diastolic volume; ESV, end systolic volume; RV, right ventricular.

In our clinical practice, congenital heart disease patients considering becoming pregnant may self‐refer or be referred for evaluation in a “pregnancy and heart disease” clinic, run jointly by a cardiologist and obstetrician from the Royal Brompton Hospital and Chelsea and Westminster Hospital pregnancy and heart disease service. TOF patients undergo prepregnancy evaluation, including CMR, to accurately assess RV size and function and quantify pulmonary regurgitation (unless performed in preceding 2–3 years and no new clinical indication). Between 2002 and 2014, all women with repaired TOF (aged between 16 and 45 years), managed by this service were identified. Electronic patient records were interrogated to confirm whether patients had a CMR scan before pregnancy and at least 6 months after delivery. Women were excluded if they did not have a complete set of imaging or if they had had a surgical intervention that would influence these measures between the interval CMR studies, such as a pulmonary valve replacement (PVR). Each pregnancy case was then matched with 2 control patients with repaired TOF from the cohort of such patients at the Royal Brompton Hospital (Figure 1). Medical records were reviewed to ensure that controls had not been pregnant. Cases were sequentially matched for age at baseline CMR scan, baseline RV end systolic volume (ESV) index, RV end diastolic volume (EDV) index, time interval, QRS duration, and RV ejection fraction using “nearest neighbor.” The study was locally registered and approved by our institution. This study was a retrospective analysis of data collected; therefore, individual informed consent was waived (UK National Health Service Health Research Authority).

### CMR Acquisition and Analysis

CMR was acquired to quantify ventricular volumes, mass, and function indexed to body surface area, in addition to pulmonary regurgitant fraction in a standardized fashion. Retrospectively gated, steady‐state free precession cine CMR of the heart was acquired in contiguous short‐axis cines covering the entirety of both ventricles using 1.5 Tesla (Siemens Healthcare, Erlangen, Germany). Typical image parameters were repetition time/echo time/ analog‐to‐digital convertor bandwidth/flip angle/slice thickness/spatial and temporal resolution: 2.6 ms/1.1 ms/930 Hz/pixel/55 degrees/7 mm/ 2.0×2.0 mm/39 ms. Twenty‐five cine frames were interpolated by retrospective gating over the patient's average RR interval during an approximately 11‐cardiac‐cycle breath‐hold. Pulmonary regurgitation fraction (% pulmonary backward flow/pulmonary forward flow) was determined using a through‐plane (velocity encoding range, velocity encoding range adjusted to suit patient) phase‐contrast gradient‐echo, breath‐hold sequence in the main pulmonary artery above the valve annulus and prebifurcation of the pulmonary arteries (repetition time/echo time/ analog‐to‐digital convertor bandwidth/flip angle/slice thickness/spatial and temporal resolution: 5.8 ms/2.8 ms/391 Hz/pixel/20 degrees/8 mm/1.5(FE)×3.0(PE)mm)/46 ms.

CMR data were analyzed by a single experienced operator blinded to whether the woman had been pregnant, and to the order of scans (G.S.), using CMRtools (Cardiovascular Imaging Solutions Ltd, London, UK). Aortic diameter was measured in diastole and systole from the coronal LV outflow tract view at the following sites: valve annulus, sinus of Valsalva, sinotubular junction, and ascending aorta at right pulmonary artery level. For volumetric analysis, a semiautomated plugin (LVtools) was utilized. Endo and epicardial surfaces were manually delineated by placing points on the cardiac borders on the short‐axis images. A threshold function allowed the operator to segment blood pool from the myocardium to include papillary muscles and trabeculations in myocardial volume. Valve attachment points were identified on the long‐axis images and tracked throughout the cardiac cycle to produce a model of the valve planes, ensuring that only ventricular blood volume and mass were used for analysis. The resultant 4 dimensional cardiac model allowed calculation of EDV, ESV, and mass using Simpson's rule for each ventricle. From these volumes, stroke volume (SV) and ejection fraction were calculated. All volume measurements were indexed for body surface area and expressed in mL/m^2^. By this method, normal indexed RV EDV is <108 mL/m^2^ and normal indexed RV ESV is <47 mL/m^2^.^8^

### Pregnancy Data

Data relating to pregnancy were collected, including gestation at delivery and type of delivery (spontaneous vaginal, assisted vaginal, and emergency Caesarean or elective Caesarean), estimated volume of blood loss at delivery and whether this constituted a postpartum hemorrhage (blood loss >500 mL at vaginal delivery or >1000 mL at Caesarean section), and birthweight and adjusted birthweight centiles for all babies.

### Statistical Analyses

STATA software 13.1 (version 13.1; StataCorp LP, College Station, TX) was used for statistical analysis and figures. Data were examined for normality using the Shapiro–Wilk test: Non‐normally distributed variables were zero‐skewness log‐transformed to ensure normal distribution before analysis. Descriptive statistics are expressed as mean (±95% CI) when normally distributed and geometric mean (±95% CI of geometric mean) when non‐normally distributed, unless specified. Proportions are expressed as percentages. Group differences in proportions were compared with a proportions test.

Effect of pregnancy on cardiac structure (ventricular and aortic root data) and exercise capacity was assessed using 2‐level linear mixed‐effects modeling. For cardiac and aortic measurements, the model setup included fixed effects: pregnancy, patient age at assessment (accounting for time interval between repeat measurements), and body surface area. For exercise data, fixed effects were pregnancy and time interval.

The primary outcome measure of the study was detection of a clinically significant increase in RV EDV index greater than 150 mL/m^2^ (conventional threshold for PVR) from baseline attributed to pregnancy. The study power was 0.77, with alpha of 0.05 (repeated measures, correlation 0.93).

Intraclass correlation coefficient was used to assess interobserver variability using a 2‐way mixed‐effects model (rater fixed, case random).

## Results

Between 2002 and 2014, 32 women with repaired TOF had a successful pregnancy. Thirteen women did not meet inclusion criteria because paired CMR data were not available: 5 did not have a prepregnancy CMR scan and 8 did not have a postpregnancy CMR scan. Of those 8 women who did not have a postpregnancy CMR scan, 5 patients had not yet undergone scheduled CMR scan examinations, 2 patients defaulted from follow‐up and 1 patient underwent elective PVR, which was scheduled before conception and undertaken 10 months after pregnancy without repeat CMR scan. This left a total of 19 women who were included in the study. These subjects were matched to 38 female controls with repaired TOF who had not been pregnant. One patient in the pregnancy and 2 matched women in the control group had previously undergone a PVR.

### Demographics

Pregnant women and nonpregnant controls were well matched for demographic characteristics at baseline (Table 1). In particular, there were no significant differences in baseline age, CMR scan interval, New York Heart Association class (all patients were New York Heart Association Class 1), TOF subtype, type or number of repairs, and QRS duration. Pregnant patients were heavier than nonpregnant controls at baseline, and this difference increased following pregnancy (*P*=0.008). The majority of patients were not taking any medications and arrhythmias were uncommon.

**Table 1:** Demographic Data for Study Participants

	Control n=38	Pregnancy n=19	*P* Value
Baseline age, y	26 (24–28)	27 (24–30)	0.7
Time interval CMR1‐CMR2, y^a^	4.1 (3.6–4.7)	4.0 (3.2–5.0)	0.8
Time interval CMR1 delivery, y^a^	···	2.1 (1.5–3.0)	···
Time interval delivery‐CMR2, y^a^	···	1.8 (1.2–2.5)	···
Weight CMR1, kg^a^	59 (55–62)	66 (60–74)	**0.03**
Weight CMR2, kg^a^	59 (56–63)	68 (62–76)	**0.008**
Body mass index CMR1	22 (20–24)	25 (22–28)	0.05
Body mass index CMR2	22 (20–24)	26 (23–29)	**0.02**
QRS duration CMR1^a^	136 (129–142)	141 (131–152)	0.07
Age at primary repair, y^a^^,^ b	2.0 (1.6–2.5)	2.8 (1.7–4.7)	0.5
RV systolic pressure, mm Hg^a^	35 (32–37)	39 (34–44)	0.1
RV outflow tract peak gradient, mm Hg^a^	6.0 (5.2–7.1)	7.1 (5.1–9.8)	0.2
New York Heart Association class I (%)	38 (100)	19 (100)	1.0
Primary diagnosis (%)			
TOF pulmonary stenosis	34 (89)	15 (79)	0.3
TOF pulmonary atresia	2 (8)	4 (21)	0.2
TOF double‐outlet RV	1 (3)	0 (0)	0.5
Repair type (%)			
Transannular patch	19 (50)	10 (53)	0.9
RV‐PA conduit	7 (18)	4 (21)	0.8
Other	12 (32)	5 (26)	0.7
Previous palliation (%)			
BT shunt	11 (29)	5 (26)	0.8
Waterston shunt	1 (3)	1 (5)	0.6
Brock procedure	1 (3)	0 (0)	0.5
Open pulmonary valvotomy	3 (8)	4 (21)	0.2
Medications (%)			
Diuretrics	1 (3)	0 (0)	0.5
ACE inhibitors	1 (3)	0 (0)	0.5
Beta‐blockers	1 (3)	1 (5)	0.6
Previous arrhythmia (%)			
Atrial arrhythmia	1 (3)	1 (5)	0.6
Ventricular arrhythmia	1 (3)	1 (5)	0.6

Data presented: mean and (95% CI of mean). ACE indicates angiotensin‐converting enzyme; BT, Blalock–Taussig; CMR, cardiovascular magnetic resonance; PA, pulmonary artery; RV, right ventricular; TOF, tetralogy of Fallot.

a Geometric mean and (95% CI of geometric mean) for non‐normally distributed data.

b Welch correction for unequal variance.

The included sample of patients was representative of the overall female population with repaired TOF in our institution in terms of RV dilatation (Figure 1).

### Pregnancy Data

All the pregnant women in the study delivered at term (37–42 weeks gestation), with a mean gestation length of 38.7 weeks. Epidurals were used for pain relief in 18 of 19 (95%). Mode of delivery was vaginal in 13 of 19 (68%), 9 of 13 (69%) of which were assisted with ventouse or forceps (4 for fetal distress, 5 because of an imposed shortened second stage). Six women had a Caesarean section, elective in 2 of 19 women (11%; 1 for breech presentation and 1 for low lying placenta). Four of 19 (21%) had an emergency Caesarean section, 2 cases for suspected fetal compromise in labor and 2 for failure to progress. Mean blood loss at delivery was 535 mL. Six (6 of 19) women had a postpartum hemorrhage: 1 at emergency caesarean section and 5 during vaginal delivery (4 of 5 assisted deliveries), the most significant being 1200 mL following a forceps delivery. Mean birthweight was 3087 g, and no babies were born below the 10th centile, corrected for gestational age and maternal demographics.

### CMR Imaging Data

Pregnant and nonpregnant controls were well balanced for baseline CMR scan data, (Table 2). In particular, there were no differences in baseline indexed RV EDV 115 (106–123 mL/m^2^) versus 118 (104–131 mL/m^2^), RV ESV 47 (42–52 mL/m^2^) versus 49 (41–57 mL/m^2^), or pulmonary regurgitation fraction 27 (19%–40%) versus 28 (14%–56%). Furthermore, there were no baseline differences detected in aortic geometry between women who later had a pregnancy and controls (Table 3). Body surface area was slightly higher in the pregnancy group compared with controls (1.7 versus 1.6 at baseline), attributed to higher weight at baseline.

**Table 2:** Ventricular CMR Data at Baseline (T1) and Follow‐up (T2)

	Control T1 (n=38)	Control T2 (n=38)	Pregnancy T1 (n=19)	Pregnancy T2 (n=19)	Fixed‐Effect Pregnancy (*P*)	Fixed‐Effect Time (*P*)
Body surface area, m^2^ a	1.6 (1.6–1.7)	1.6 (1.6–1.7)	1.7 (1.7–1.8)	1.8 (1.7–1.8)	**0.01**	**0.002**
Weight, kg^a^	58 (55–62)	59 (55–62)	66 (60–74)	68 (62–75)	**0.02**	**0.001**
RV EDV index, mL/m^2^ a	115 (106–123)	116 (108–125)	118 (104–131)	119 (103–138)	0.51	**0.02**
RV ESV index, mL/m^2^ a	47 (42–52)	46 (42–51)	49 (41–57)	49 (41–60)	0.40	0.12
RV SV index, mL/m^2^ a	67 (62–72)	70 (65–76)	68 (61–75)	71 (62–80)	0.77	**0.01**
RV ejection fraction, %	59 (56–61)	60 (57–62)	59 (56–62)	58 (55–61)	0.48	0.84
RV mass index, g/m^2^	49 (46–52)	50 (47–53)	50 (45–54)	52 (46–58)	0.39	0.34
Pulmonary regurgitant fraction, %^a^	29 (1–49)	34 (1–55)	26 (1–50)	28 (5–52)	0.22	**0.002**
LV EDV index, mL/m^2^ a	76 (72–80)	76 (72–80)	84 (75–94)	84 (75–94)	**0.03**	0.99
LV ESV index, mL/m^2^ a	27 (25–29)	28 (26–30)	30 (26–34)	30 (26–35)	0.13	0.46
LV SV index, mL/m^2^ a	49 (46–51)	49 (46–51)	53 (47–60)	55 (49–61)	**0.04**	0.73
LV ejection fraction, %	64 (62–66)	64 (63–65)	64 (61–67)	65 (63–68)	0.88	0.13
LV mass index (g/m^2^)^a^	53 (51–56)	54 (52–56)	59 (53–65)	59 (53–66)	0.05	0.70

Data presented as mean and (95% CI of mean). CMR indicates cardiovascular magnetic resonance; EDV, end diastolic volume; ESV, end systolic volume; LV, left ventricular; RV, right ventricular; SV, stroke volume.

a Geometric mean and (95% CI of geometric mean) for non‐normally distributed data.

**Table 3:** Aortic Geometry at Baseline (T1) and Follow‐up (T2)

	Control T1 (n=38)	Control T2 (n=38)	Pregnancy T1 (n=19)	Pregnancy T2 (n=19)	Fixed‐Effect Pregnancy (*P*)	Fixed‐Effect Time (*P*)
Aortic valve annulus diastole^a^	25.2 (24.4–26.2)	25.3 (24.4–26.2)	25.4 (23.6–27.4)	25.5 (23.7–27.4)	0.87	0.39
Aortic valve annulus systole^a^	23.7 (22.7–24.8)	23.6 (22.6–24.6)	24.0 (22.0–26.2)	24.2 (22.3–26.3)	0.81	0.93
Sinuses of valsalva diastole^a^	31.3 (30.1–32.5)	32.0 (30.8–33.2)	32.7 (30.6–35.0)	33.0 (30.9–35.3)	0.34	**<0.0005**
Sinuses of valsalva systole	32.6 (31.2–34.0)	32.6 (31.3–34.0)	33.9 (31.7–36.2)	34.0 (31.6–36.4)	0.43	0.42
Sinotubular junction diastole^a^	26.6 (25.1–28.2)	27.5 (26.1–29.0)	27.4 (24.3–30.9)	27.9 (24.9–31.3)	0.75	**<0.0005**
Sinotubular junction systole^a^	28.6 (27.1–30.1)	28.7 (27.1–30.2)	29.1 (26.1–32.3)	29.0 (25.9–32.5)	0.86	0.25
Ascending aorta diastole^a^	25.5 (24.0–26.9)	26.4 (25.2–27.7)	26.5 (23.7–29.7)	27.6 (24.6–31.0)	0.53	**<0.0005**
Ascending aorta systole^a^	28.0 (26.7–29.5)	28.3 (27.2–29.5)	28.7 (26.2–31.5)	29.8 (26.8–33.0)	0.61	**<0.0005**

Data are expressed as diameter (mm): mean and (95% CI of mean).

a Geometric mean and (95% CI of geometric mean) for non‐normally distributed data.

### Ventricular Data

**Figure 2:**
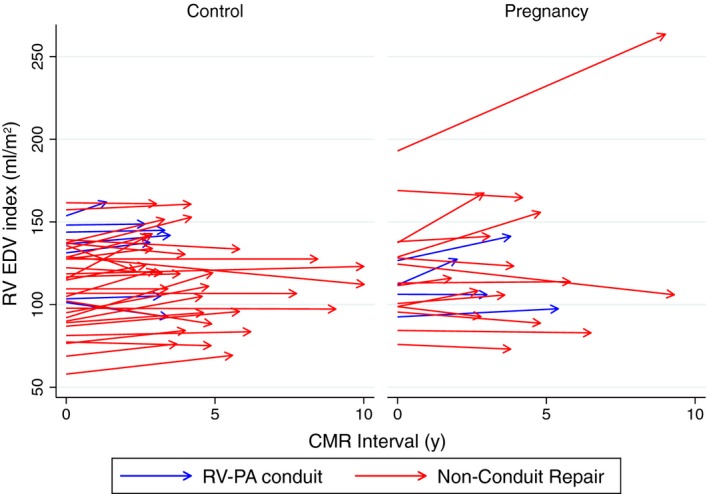
RV EDV index (mL/m^2^) at baseline (time=0) and at follow‐up in control and pregnancy groups; patients with an RV‐PA conduit repair are shown in blue. EDV indicates end diastolic volume; PA, pulmonary artery; RV, right ventricular.

**Figure 3:**
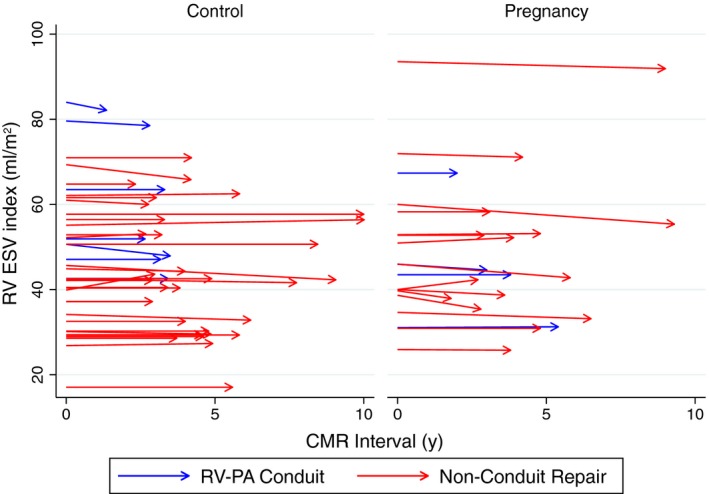
RV ESV index (mL/m^2^) at baseline (time=0) and at follow‐up in control and pregnancy groups; patients with an RV‐PA conduit repair are shown in blue. ESV indicates end systolic volume; PA, pulmonary artery; RV, right ventricular.

We detected no effect of pregnancy on RV parameters between groups from baseline to follow‐up (Table 2; Figures 2 and 3). As expected, there were small increases in RV EDV, RV SV, and PR over time, which were not potentiated by pregnancy.

In contrast, there was an effect of pregnancy observed in both LV EDV and LV SV, consistent with a sustained small increase in LV SV attributed to pregnancy (53–55 mL/m^2^). There was also a trend toward a pregnancy effect in LV Mass (*P*=0.05).

The pregnant patient with the highest baseline RV size (RV EDV 193 mL/m^2^ and RV SV 94 mL/m^2^) was previously lost to follow‐up and represented to cardiology care not long before conceiving. This patient also experienced the largest interval change in RV EDV 264 mL/m^2^ and RV ESV 146 mL/m^2^. We would ordinarily recommend PVR at this level of RV dilatation before pregnancy.

### Aortic Root

We observed no pregnancy effect on aortic root geometry at all levels (annulus, sinuses, sinotubular junction, and ascending aorta; Table 3). However, as expected, there were small increases in diastolic aortic dimensions (sinuses of valsalva, sinotubular junction, and ascending aorta) with time in both groups and in the systolic ascending aorta dimension. However, the mean group change in these parameters was 1 mm or less over 4 years and were not potentiated by pregnancy.

### Exercise Testing

Exercise data were available for 26 of 38 (68%) controls and 13 of 19 (68%) pregnant patients at baseline and 28 of 38 (72%) controls and 14 of 19 (74%) pregnant patients at follow‐up (Table 4). We observed no effect of pregnancy on any exercise parameter. There was an effect of time on resting heart rate, with an increase in resting heart rate at the follow‐up test.

**Table 4:** Exercise at baseline (T1) and Follow‐up (T2)

	Control T1 (n=26)	Control T2 (n=28)	Pregnancy T1 (n=13)	Pregnancy T2 (n=14)	Fixed‐Effect Pregnancy (*P*)	Fixed‐Effect Interval (*P*)
Age at exercise test, y	26 (24–29)	30 (27–34)	26 (24–29)	30 (27–34)	—	—
Resting heart rate, bpm	79 (75–83)	82 (77–87)	82 (76–88)	82 (77–86)	0.57	**0.01**
Maximum heart rate, bpm^a^	169 (161–178)	170 (163–178)	174 (167–182)	169 (162–176)	0.87	0.16
RER^a^	1.1 (1.1–1.2)	1.1 (1.1–1.1)	1.1 (1.1–1.2)	1.1 (.98–1.2)	0.81	0.38
VO_2_ peak, mL/min/kg	24 (21–26)	26 (24–29)	25 (20–30)	25 (20–29)	0.87	0.16
Predicted VO_2_, %	72 (65–79)	82 (75–88)	79 (69–89)	79 (67–91)	0.46	0.06
VO_2_ at AT, mL/min/kg	16 (14–18)	18 (17–20)	15 (13–18)	17 (14–21)	0.46	**0.04**
VE/VCO_2_ slope^a^	30 (28–32)	29 (27–32)	32 (27–37)	32 (29–36)	0.11	0.93

Data are expressed as: mean and (95% CI of mean). AT indicates anaerobic threshold, RER, respiratory exchange ratio, VE/VCO_2_, Ventilatory equivalent ratio for carbon dioxide; VO_2_, oxygen consumption.

a Geometric mean and (95% CI of geometric mean) for non‐normally distributed data. Fixed effects, pregnanc,y and time interval.

### Interobserver Variability

Volumetric measurements were made in a random subset of 7 patients by a blinded second observer (E.L.H.) for the assessment of interobserver variability. There was good interobserver reproducibility as assessed by intraclass correlation coefficients (Table 5)

**Table 5:** ICCs for Interobserver Variability 2‐Way Mixed‐Effects Model (Rater Fixed, Case Random Effects)

Parameter	ICC (95% CI)	Probability, *P*
LV end diastolic volume	0.95 (0.73–0.99)	*P*<0.0005
LV end systolic volume	0.96 (0.79 –0.99)	*P*<0.0005
LV stroke volume	0.88 (0.47–0.98)	*P*=0.002
LV ejection fraction	0.88 (0.46–0.98)	*P*=0.002
LV mass	0.98 (0.91–1.0)	*P*<0.0005
RV end diastolic volume	0.95 (0.73–0.99)	*P*<0.0005
RV end systolic volume	0.74 (0.07–0.95)	*P*=0.02
RV stroke volume	0.95 (0.74–0.99)	*P*<0.0005
RV ejection fraction	0.57 (0–0.91)	*P*=0.07
RV mass	0.97 (0.83–0.99)	*P*<0.0005

EDV indicates end diastolic volume; ESV, end systolic volume; ICC, intraclass correlation coefficients; LV, left ventricular; RV, right ventricle; SV, stroke volume.

## Discussion

Our study indicates that pregnancy is not associated with a significant deterioration in RV volume or worsening of pulmonary regurgitation in women with mild‐to‐moderate baseline RV dilatation. Furthermore, we also observed no effect of pregnancy on either aortic dimensions or exercise testing. There was a small effect of pregnancy on LV EDV and LV SV, consistent with a small and sustained increase in these metrics following pregnancy.

These data are important because they refine our understanding about the effects of pregnancy in women with moderate or better RV dilatation, an area hitherto understudied. Prepregnancy counseling is made difficult by a paucity of data, and has tended toward the view that the cardiovascular adaption to pregnancy is impaired in women with structural heart disease.^9^, ^10^, ^11^

Our data, regarding lack of significant and progressive deterioration in RV volumes associated with pregnancy in women with repaired TOF, contrast with the only previous study to investigate pregnancy in this population using CMR.^5^ These disparities may be explained, at least in part, by important baseline differences between the 2 study populations. Egidy Assenza et al reported that, compared with nonparous controls, their pregnant patients had accelerated deterioration in RV dimensions: mean RV EDV index increased from 135 mL/m^2^ at baseline to 147 mL/m^2^ postpregnancy. Despite being of similar age at baseline CMR and at primary repair, the patients we report on in our current study had a much lower mean prepregnancy RV EDV index of 118 mL/m^2^ and the value after pregnancy of 119 mL/m^2^ was not significantly greater. In a small subgroup analysis, Egidy Assenza et al suggested that the patients with the worst initial RV dilatation (>152 mL/m^2^) were those who experienced greatest progression. Data from both studies may therefore be viewed as complementary, indicating that patients with milder RV dilatation are less likely to experience significant deterioration as a consequence of pregnancy. This important and reassuring clinical information may help to refine prepregnancy counseling in this population.

Pregnancy is associated with an increased risk of aortic events in women with connective tissue disease. Although aortic dilatation is common in repaired TOF, it is associated with a very low risk of dissection/rupture. While the possibility of unexpected dilatation of the aorta during pregnancy exists, our data do not support this view and add further information in support of the low‐risk nature of aortic root dilatation in TOF.

Our study identified a persistent small increase in LV EDV and LV SV and trend increase in LV mass following pregnancy. These data are consistent with our understanding of normal LV adaptation following pregnancy.^12^ These changes may be related to the increased physiological and physical demands of motherhood and are not expected to be limited to the TOF population, although a case control study comparing whether LV adaptation in TOF is similar to healthy pregnant patients would be useful.

The favorable obstetric outcomes observed in our pregnancy cohort may also reflect the overall cardiovascular health of the pregnancy group. Our previous study of women with repaired TOF showed a higher rate of small for gestational age babies (35% had a birthweight <10th centile) than healthy women.^13^ In this later cohort, no babies were less than the 10th centile, although 68% (13 of 19) were below the 50th centile.

Our sample is typical of young female TOF patients in their third postoperative decade currently seen in our institution. These data suggest a cautious optimism for the majority of women contemplating pregnancy, especially those with mild‐to‐moderate RV dilatation. It is important to note that clinical practice with regard to the management of pulmonary regurgitation and RV dilatation after repair of TOF has changed markedly over the last decade(s) and that historic prepregnancy advice will need to be regularly updated in order to reflect the current disease spectrum and evolving clinical practice.

Important questions remain in this group, including the performance of pulmonary homografts during pregnancy and further stratification of patients to determine whether there are acceptable levels of progressive RV dilatation in those patients with a larger prepregnancy RV. Future larger, prospective studies may shed light on this. For the time being, we continue to observe indications for PVR suggested for all repaired TOF patients, including using contemporary consensus preoperative CMR RV volume cutoffs for optimal timing of PVR (RV volumes of ≈80–90 mL/m^2^ and 150–160 mL/m^2^ in systole and diastole, respectively).^14^, ^15^, ^16^ Based on the Egidy Assenza et al data, we would have greater concern for those patients with prepregnancy levels of RV dilatation, especially those approaching levels of dilatation qualifying for PVR. These data emphasize that women of reproductive age require proactive and tailored prepregnancy counseling^17^ and also require a rigorous approach to regular follow‐up CMR imaging postpregnancy.^18^

## Limitations

The current study is limited by its relatively small sample size and retrospective study design in a single center; it is therefore not powered to detect very small differences in parameters. To overcome the limitations of small sample size, we utilized a matched control group and statistical analysis using mixed‐effects modeling to leverage repeated measures. An important limitation of our retrospective matching technique is that it will not prevent bias attributed to other unmatched variables.

Because of the organization of adult congenital heart disease and pregnancy services in the United Kingdom, we believe that the sample is representative of pregnancy in repaired TOF in the current era. Ideally, all patients would have had baseline CMR imaging within a year of pregnancy; but we know many women do not plan a pregnancy and or may take several years to conceive, which may make this difficult to achieve.

## Conclusion

There is no significant longitudinal change in measurements of the RV or aorta associated with pregnancy in repaired TOF with mild‐to‐moderate RV dilatation when compared with controls. Changes in LV dimensions are consistent with normal pregnancy adaptation. Our conclusions are based on a relatively healthy group of women and may not apply to women with the most severe RV dilatation; however, the group included in this study is representative of the overall contemporary population of women of childbearing years with repaired TOF in our center. Women with mild‐to‐moderate RV dilatation considering pregnancy can therefore be reassured that pregnancy is unlikely to cause a deterioration in their disease status as a consequence. The contemporary cohort of women of childbearing age with repaired TOF may have lower pregnancy‐related risks than previously described cohorts undergoing pregnancy. Prepregnancy counseling and clinical management in women with repaired TOF can be tailored to their individual clinical status.

## Sources of Funding

Babu‐Narayan is supported by an Intermediate Clinical Research Fellowship from the British Heart Foundation (FS/11/38/28864). Michael Quail is supported by a British Heart Foundation‐Fulbright Scholarship (FS/16/28/32327). This project was supported by the NIHR Cardiovascular Biomedical Research Unit of Royal Brompton and Harefield NHS Foundation Trust and Imperial College London. This report is independent research by the National Institute for Health Research Biomedical Research Unit Funding Scheme. The views expressed in this publication are those of the author(s) and not necessarily those of the NHS, the National Institute for Health Research, or the Department of Health.

## Disclosures

None.
